# Long-term outcome of Willis covered stent for direct carotid-cavernous fistula

**DOI:** 10.3389/fneur.2026.1753105

**Published:** 2026-01-22

**Authors:** Tao Zhang, Hu Chen, Yufeng Liu, Wei Fang, Guoqiang Luo, Xing Guo, Haiyang Jiang, Zijian Yang, Yue Si, Zhenwei Zhao, Jianping Deng

**Affiliations:** Department of Neurosurgery, Tangdu Hospital, Air Force Medical University, Xi’an, China

**Keywords:** direct carotid-cavernous fistula, endovascular therapy, long-term outcome, retrospective study, Willis covered stent

## Abstract

**Objective:**

This retrospective single-center study aimed to evaluate the safety, efficacy, and long-term outcomes of the Willis covered stent (WCS) in the treatment of direct carotid-cavernous fistula (dCCF).

**Methods:**

Between November 2014 and November 2019, 13 patients with dCCF (out of 66 eligible cases) were enrolled and treated with WCS in our institution. Clinical characteristics, procedural details and follow-up data were collected and analyzed.

**Results:**

Technical success in WCS delivery and deployment was achieved in all 13 patients. No device-related or procedure-related complications were observed. Postoperative angiography demonstrated complete fistula occlusion in 10 patients (76.9%), minimal endoleak in 2 patients (15.4%), and minimal residual leakage in 1 patient (7.7%). Cranial bruit was resolved in all affected patients immediately after the procedure. Proptosis completely resolved in all 13 patients (100%) at the 1-month clinical follow-up (FU). During angiographic FU (mean duration: 9 ± 5.9 months), complete fistula occlusion and parent artery patency were maintained in all 13 patients (100%), with no recurrence of dCCF or in-stent stenosis detected. Long-term clinical FU data were available for 12 patients (92.3%), with a mean duration of 76.8 ± 29.7 months. Ocular symptoms fully resolved in 9 patients (75%), while persistent visual decline was noted in 1 patient (8.3%) and permanent visual loss with mild ptosis was observed in 2 patients (16.7%).

**Conclusion:**

Our findings demonstrated that WCS was a safe and effective therapeutic option for the treatment of dCCFs, with favorable long-term clinical outcomes. However, additional large-scale studies and prospective randomized controlled trials are warranted to validate these results.

## Introduction

Direct carotid-cavernous fistulas (dCCFs) are characterized by a pathological arteriovenous shunt between internal carotid artery (ICA) and cavernous sinus (CS) (1). Based on etiology, dCCFs can be classified into traumatic (80%), spontaneous, and iatrogenic subtypes (2). The most common anatomical locations of dCCFs were reported to be the proximal horizontal portion and posterior bend of the cavernous ICA (3, 4). The shunting blood flow in dCCFs might induce venous hyperperfusion, manifesting as diverse clinical symptoms including proptosis, cranial bruit, diplopia, and visual impairment.

The optimal treatment goal for dCCFs is complete occlusion of the fistulous rent, while preserving ICA patency and minimizing compromise of the CS. Endovascular therapy (EVT) has become the first-line treatment since Serbinenko first treated the dCCF using a detachable balloon in 1974 (5). Compared with other EVT modalities, such as detachable balloons (DBs), coils, liquid embolic agent, or flow diverters (FDs), the Willis covered stent (WCS, MicroPort, Shanghai, China) offers several distinct advantages: (i) immediate fistula occlusion via parent artery reconstruction without intraluminal mass protrusion; (ii) preservation of CS drainage to reduce the risk of cranial nerve palsy; and (iii) simplified, time-efficient procedures that avoid intra-fistulous manipulation (6).

Several retrospective studies have evaluated the safety and efficacy of WCS for dCCF treatment with promising results (7, 8). However, these studies were limited by small sample sizes and a lack of long-term outcome data. Additional evidence regarding clinical outcomes and technical nuances of this EVT modality is needed. Therefore, the present retrospective single-center study aimed to report our experience with WCS for dCCF treatment, with a focus on long-term outcomes.

## Materials and methods

### Patient selection

A total of 66 patients with dCCFs were identified from the “Database of Cerebrovascular Diseases in Tangdu Hospital” between November 2014 and November 2019. Eligibility for WCS treatment was independently evaluated by two senior interventional neuroradiologists based on angiographic characteristics, including fistula location, parent artery diameter, and vessel tortuosity, with the following criteria: (i) with respect to the carotid siphon, fistula location was proximal to the anterior bend of the cavernous ICA; (ii) maximum parent artery diameter ≤ 4.5 mm; (iii) the size of fistulous rent ≤ 12 mm (considering the maximum available WCS length of 16 mm, with a minimum of 2 mm healthy vessel coverage required on both sides of the lesion); and (iv) posterior bend angle of the cavernous ICA ≥ 90° (9). Alternative EVT strategies, including DBs, coils, liquid embolic agents or FDs, were discussed with all patients and their relatives. Ultimately, 13 patients with dCCFs were enrolled in the study. Clinical data, stent specifications, procedural details, and postoperative/follow-up (FU) results were collected and analyzed.

This study was approved by the Ethics Committee of Tandu Hospital. Written informed consent was obtained from each patient for the purpose of research.

### Segments of the cavernous ICA

The cavernous ICA, which extends from the superior margin of the petrolingual ligament to the proximal dural ring, is divided into four segments along the direction of blood flow: vertical portion, posterior bend, horizontal portion and anterior bend (4). The vertical and horizontal portion are more favorable for covered stent placement than the posterior and anterior bends.

### Localization of fistulous rent

Fistula localization might be challenging in high-flow dCCF cases. Therefore, supplementary angiographic evaluations should be performed, including vertebral artery angiography (lateral view) and contralateral carotid artery angiography (anterior–posterior view) with/without ipsilateral carotid compression. Furthermore, three-dimensional (3D) rotational angiography would be practical and effective. The site of fistulous rent could be identified by tracing the ICA wall on multiplanar reconstruction (MPR) images orthogonal to the suspected lesion, where a “broken-rim sign” (discontinuity or indistinctness of the vessel wall) indicated the rent. The size of fistulous rent could be measured on the same MPR views (10).

### Endovascular procedures

Endovascular procedures were performed under general anesthesia using a Siemens Artis Zee biplane angiographic system (Siemens, Munich, Germany). Intravenous heparinization was administered to maintain an activated clotting time of 250–300 s.

For non-tortuous vascular pathways, a 6Fr Envoy guiding catheter (Codman, Raynham, MA, USA) was used to deliver the WCS via a 0.014-inch Synchro microguidewire (Stryker, Kalamazoo, MI, USA). For tortuous pathways, a coaxial system was employed, consisting of an 8Fr Envoy guiding catheter (Codman, Raynham, MA, USA) or a 6Fr NeuroMax long sheath (Penumbra, Alameda, CA, USA), a 5F or 6F Navien Intracranial Support Catheter (Covidien, Mansfield, MA, USA), and an XT-27 microcatheter (Stryker, Kalamazoo, MI, USA). In this scenario, the Navien catheter was advanced across the fistula, followed by withdrawal of the XT-27 microcatheter. The WCS (housed within the Navien catheter) was positioned at the target site, and then the Navien catheter was slowly withdrawn to expose the stent for deployment. Under roadmap monitoring, the WCS was deployed by inflating the balloon at an appropriate pressure. Immediate post-deployment angiography was performed to assess stent apposition and fistula occlusion.

Endoleak could be inevitably encountered after initial stent deployment owning to the diameter gradients along the covered artery. Therefore, balloon re-inflation (*in situ*, proximally, or distally) with moderately increased pressure should be performed to eliminate endoleak. Persisted endoleak after several balloon re-inflation (≤ 3 times) should be discriminated and managed based on severity. Minimal endoleak in the middle-to-distal segment of the WCS could be monitored conservatively, while significant endoleak or residual fistulous flow should be promptly addressed with additional WCS deployment or alternative EVT strategies. Postoperative angiography, physical examination, and computed tomography (CT) were routinely performed to rule out procedure related complications.

### Antithrombotic management

Preoperatively, patients received dual antiplatelet therapy (DAPT) with aspirin (100 mg/day) and clopidogrel (75 mg/day) for 3–5 consecutive days. For patients with clopidogrel resistance, ticagrelor (90 mg, twice, daily) was substituted. Emergency patients received intravenous tirofiban immediately after stent deployment (10 μg/kg bolus within 3 min, followed by 0.15 μg/kg/min maintenance infusion for 48 h), with DAPT initiated 6 h before tirofiban withdrawal. All patients were instructed to continue DAPT for 6 months and aspirin for at least 1 year.

### Clinical and angiographic follow-up

Clinical FU was conducted at discharge and 1 month postoperatively. The final clinical FU was performed via telephone interview or outpatient visit. Digital subtraction angiography (DSA) or CT angiography (CTA) was recommended for all patients to assess angiographic outcomes.

## Results

### General characteristics

Thirteen patients, 7 females (53.8%) and 6 males (46.2%), with a mean age of 50.85 ± 10.09 years (range: 25–64 years) were enrolled in our study. The etiology was traumatic (76.9%) and spontaneous (23.1%). The most common ocular symptoms were proptosis (100%), followed by cranial bruit (76.9%), diplopia (38.5%), visual decline (15.4%) and visual loss (15.4%). The sites of fistulous rent were at the vertical portion (61.5%), posterior bend (23.1%) and horizontal portion (15.4%) of the cavernous ICA, respectively. The maximum diameter of the parent artery was 4.24 ± 0.20 mm. The size of fistulous rent was 3.43 ± 0.77 mm. Detailed baseline characteristics are summarized in Table 1.

**Table 1:** Detailed baseline characteristics of the 13 patients with dCCF treated by the WCS.

Variables	*n* or mean ± SD	%
Sex		
Male	6	46.2%
Female	7	53.8%
Age (years)	50.85 ± 10.09	
Etiology		
Traumatic	10	76.9%
Spontaneous	3	23.1%
Ocular symptoms		
Proptosis	13	100%
Cranial bruit	10	76.9%
Diplopia	5	38.5%
Visual decline	2	15.4%
Visual loss	2	15.4%
Site of fistulous rent		
Vertical portion	8	61.5%
Posterior bend	3	23.1%
Horizontal portion	2	15.4%
Maximum diameter of the parent artery (mm)	4.24 ± 0.20	
Size of fistulous rent (mm)	3.43 ± 0.77	

dCCF, direct carotid-cavernous fistula. WCS, Willis covered stent.

### Procedural details and immediate outcomes

Technical success (successful WCS deployment with parent artery preservation) was achieved in all 13 patients (100%). No device-related or procedure-related complications were observed. Postoperative angiography demonstrated complete fistula occlusion in 10 patients (76.9%), minimal endoleak in 2 patients (15.4%), and minimal residual leakage in 1 patient (7.7%).

Immediately after initial WCS deployment, complete occlusion was achieved in 6 patients (46.2%), endoleak was detected in 6 patients (46.2%), and significant distal leakage was noted in 1 patient (7.7%). Balloon re-inflation was performed for all endoleak cases, resulting in complete resolution in 4 patients and persistent minimal endoleak in 2 patients (one in the middle segment and one in the distal segment of the WCS). The patient with significant distal leakage (inadequate stent coverage) underwent rescue embolization with coils and Onyx, with minimal residual leakage remaining postoperatively. All minimal endoleaks and residual leakage were carefully evaluated and deemed acceptable for conservative monitoring and FU. Detailed procedural outcomes were summarized in Tables 2, 3.

**Table 2:** Operative and follow-up results of the 13 patients with dCCF treated by the WCS.

Variables	*N* or mean ± SD	%
Technical success	13	100%
Procedural details		
WCS	12	92.3%
Stenting only	6	46.2%
Stenting + balloon re-inflation (for endoleak)	6	46.2%
WCS + Coils + Onyx	1	7.7%
Immediate post-operative result		
Complete occlusion	10	76.9%
Minimal endoleak	2	15.4%
Middle section of WCS	1	7.7%
Distal section of WCS	1	7.7%
Minimal residual leakage	1	7.7%
Procedural complications	0	0
Angiographic follow-up		
Patients	13	100%
Months	9.00 ± 5.87	
Complete occlusion	13	100%
Parent artery patency	13	100%
1-month clinical follow-up		
Patients	13	100%
No symptom	8	61.5%
Mild Diplopia	1	7.7%
Visual decline	2	15.4%
Visual loss with moderate ptosis	2	15.4%
Last clinical follow-up		
Patients	12	92.3%
Months	76.80 ± 29.72	
No symptom	9	75%
Visual decline	1	8.3%
Visual loss with mild ptosis	2	16.7%

dCCF, direct carotid-cavernous fistula. WCS, Willis covered stent.

**Table 3:** Clinical data, stent size, procedure and follow-up details of the 13 patients with dCCF treated by the WCS.

No	Sex	Age	Etiology	Ocular symptoms	Site of fistula	Stent size	Procedure details	Post-op outcome	Angiographic FU		1-month clinical FU	Last clinical FU	
									Duration	Results		Duration	Results
1	M	25	Traumatic	Proptosis, visual loss	Vertical portion	4.0 × 16 mm	Stenting only	Complete occlusion	7 months	Complete occlusion	Moderate ptosis, Visual loss	132 months	Mild ptosis, Visual loss
2	F	58	Traumatic	Proptosis, visual decline	Vertical portion	4.5 × 16 mm	proximal and distal balloon re-inflation	Complete occlusion	6 months	Complete occlusion	visual decline	112 months	No symptom
3	F	54	Spontaneous	Proptosis, bruit	Vertical portion	4.5 × 13 mm	Proximal balloon re-inflation	Complete occlusion	6 months	Complete occlusion	No symptom	109 months	No symptom
4	F	57	Traumatic	Proptosis, bruit, diplopia	Vertical portion	4.5 × 16 mm	Proximal and distal balloon re-inflation	Minimal endoleak*	8 months	Complete occlusion	No symptom	96 months	No symptom
5	M	60	Traumatic	Proptosis, bruit, diplopia	Horizontal portion	4.5 × 13 mm	*In situ* balloon re-inflation	Complete occlusion	6 months	Complete occlusion	No symptom	85 months	No symptom
6	M	64	Traumatic	Proptosis, bruit, diplopia	Posterior bend	4.5 × 13 mm	Stenting only	Complete occlusion	15 months	Complete occlusion	No symptom	77 months	No symptom
7	F	50	Spontaneous	Proptosis, visual loss	Posterior bend	4.5 × 16 mm	*In situ* balloon re-inflation	Minimal endoleak*	2 months	Complete occlusion	Moderate ptosis, Visual loss	64 months	Mild ptosis, Visual loss
8	F	46	Traumatic	Proptosis, bruit	Horizontal portion	4.5 × 13 mm	*In situ* balloon re-inflation	Complete occlusion	10 months	Complete occlusion	No symptom	NA	NA
9	M	50	Traumatic	Proptosis, bruit, diplopia	Vertical portion	4.5 × 13 mm	Stenting only	Complete occlusion	12 months	Complete occlusion	No symptom	55 months	No symptom
10	M	58	Traumatic	Proptosis, bruit, visual decline	Vertical portion	4.5 × 13 mm	Stenting only	Complete occlusion	6 months	Complete occlusion	Visual decline	49 months	Visual decline
11	M	41	Traumatic	Proptosis, bruit	Vertical portion	4.5 × 13 mm	Stenting only	Complete occlusion	5 months	Complete occlusion	No symptom	48 months	No symptom
12	F	52	Spontaneous	Proptosis, bruit, diplopia	Vertical portion	4.5 × 10 mm	Distal endoleak Coils + Onyx	Minimal leakage	25 months	Complete occlusion	Mild diplopia	48 months	No symptom
13	F	46	Traumatic	Proptosis, bruit	Posterior bend	4.5 × 16 mm	Stenting only	Complete occlusion	9 months	Complete occlusion	No symptom	47 months	No symptom

dCCF, direct carotid-cavernous fistula. F, female; FU, follow-up; M, male. NA, not applicable. WCS, Willis covered stent. *Middle segment of the stent, distal segment of the stent.

### Angiographic follow-up results

**Figure 1:**
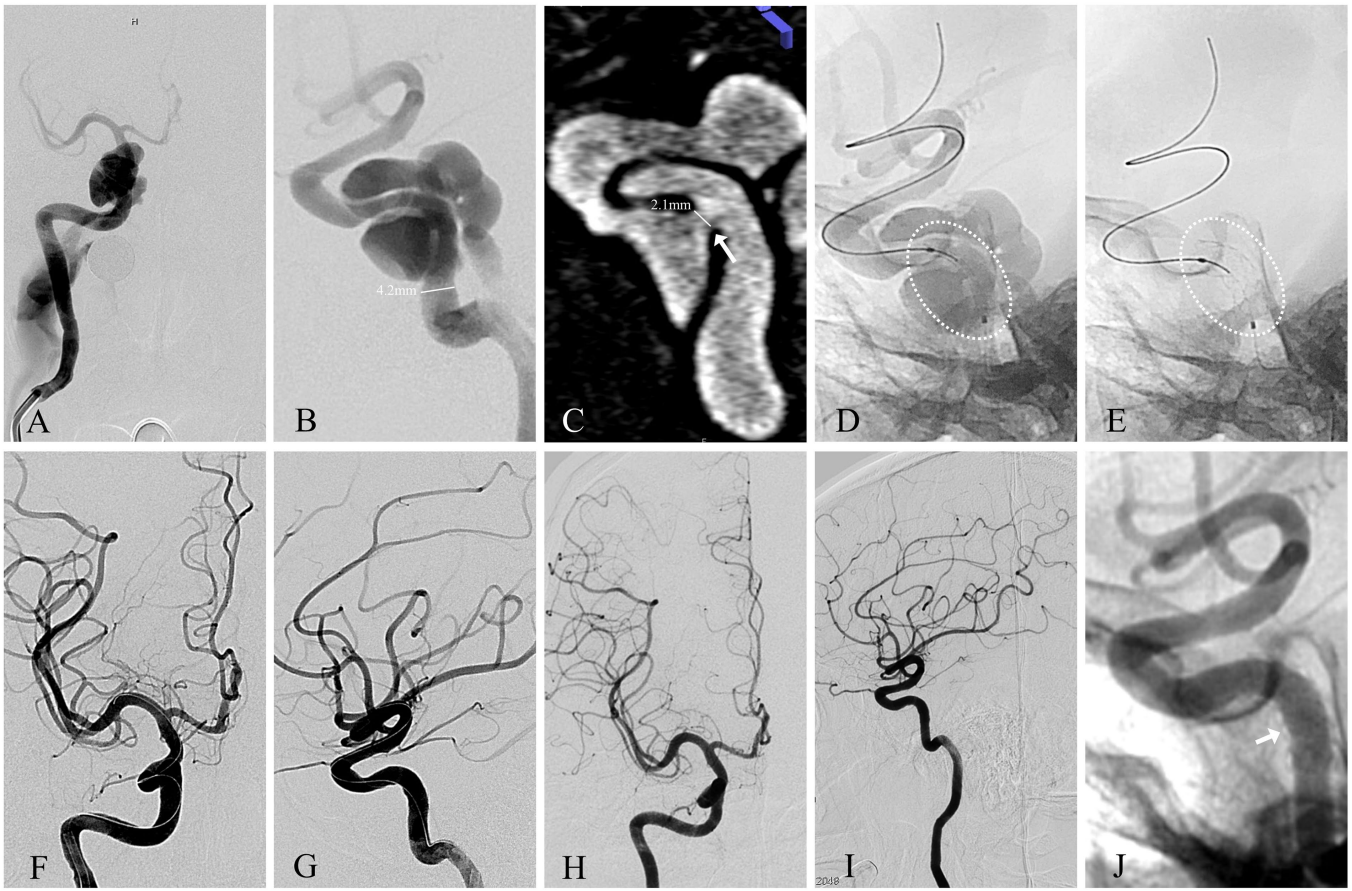
Angiographic images of a 64-year-old man with a right traumatic dCCF (see Patient No. 6 in Table 3). **(A)** A-P view and **(B)** Lateral view of a right-sided traumatic dCCF and the diameter of the parent artery (straight line). **(C)** The site (arrowhead) and size (straight line) of the fistulous rent was identified at the posterior bend of the cavernous ICA in multiplanar reconstructions (MPR) imaging orthogonal to the rent. **(D)** The WCS (dotted oval) 4.5 × 13 mm was delivered in position to cover the fistula. **(E)** The WCS (dotted oval) was deployed after initial balloon inflation. **(F)** A-P view and **(G)** lateral view of the complete occlusion of the dCCF and the preservation of the parent artery. **(H)** A-P view and **(I)** lateral view of the complete occlusion of the dCCF at the 15-month angiographic FU. **(J)** The neointimal hyperplasia (arrowhead) within the WCS.

**Figure 2:**
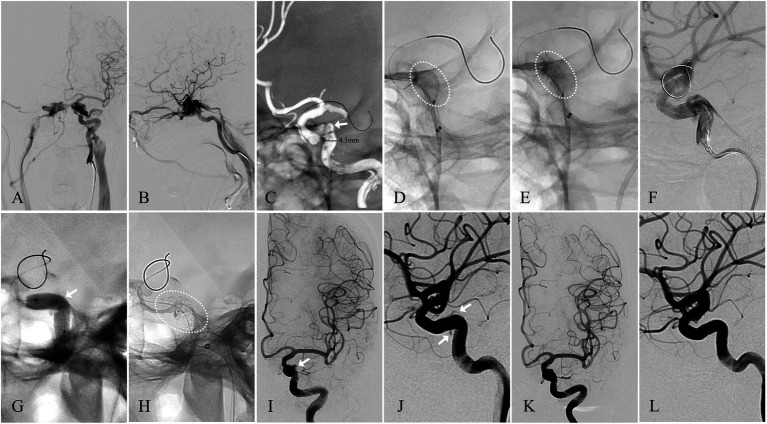
Angiographic images of a 50-year-old woman with a left-sided spontaneous dCCF (see Patient No. 7 in Table 3). **(A)** A-P view and **(B)** Lateral view of a left-sided traumatic dCCF. **(C)** The site of the fistulous rent (arrowhead) was identified at the posterior bend of the cavernous ICA by 3D reconstruction, the microguidewire was navigated by 3D roadmap, and the diameter of the parent artery (straight line) was measured. **(D)** The WCS (dotted oval) 4.5 × 16 mm was delivered in position to cover the fistula. **(E)** The WCS (dotted oval) was deployed after initial balloon inflation. **(F)** Endoleak persisted after initial balloon inflation. **(G)** Balloon re-inflation with an appropriately increased pressure. **(H)** The WC’S after balloon re-inflation. **(I)** A-P view and **(J)** lateral view of the minimal endoleak (arrowhead) at the distal section of the WCS. **(K)** A-P view and **(L)** lateral view of the complete elimination of the endoleak at the 2-month angiographic FU.

Angiographic FU data were available for all 13 patients with a mean duration of 9.00 ± 5.87 months (range: 2–25 months). Complete fistula occlusion and parent artery patency were maintained in all patients (100%), with no recurrence of dCCF or in-stent stenosis detected. Neointimal hyperplasia was observed in 1 patient at the 15-month FU (Figure 1). Notably, the 2 cases of minimal endoleak and 1 case of residual leakage resolved spontaneously during FU: the endoleak in the middle segment of the stent resolved at 8 months (patient No. 4), the endoleak in the distal segment of the stent resolved at 2 months (patient No. 7, Figure 2), and the residual leakage (after coil/Onyx rescue embolization) resolved at 25 months (patient No. 12).

### Clinical follow-up results

Cranial bruit resolved immediately after procedure in all affected patients, and all ocular symptoms except visual impairment improved gradually by discharge. At the 1-month FU, proptosis had completely resolved in all 13 patients (100%), and other ocular symptoms had fully recovered in 8 patients (61.5%). Mild diplopia persisted in 1 patient (7.7%) who had undergone coil/Onyx rescue embolization. Visual decline in 2 patients (15.4%) and visual loss in 2 patients (15.4%) showed no improvement, with moderate ptosis observed in the 2 patients with visual loss.

Long-term clinical FU data were available for 12 patients with a mean duration of 76.80 ± 29.72 months (range: 47–132 months). No strokes or new neurological deficits occurred during FU. Ocular symptoms had fully resolved in 9 patients (75%), while persistent visual decline was noted in 1 patient (8.3%) and permanent visual loss with mild ptosis persisted in 2 patients (16.7%).

## Discussion

### WCS and other EVT modality

EVT has become the cornerstone of dCCF management, driven by technological progress and material innovations in neurointervention. Although DBs pioneered dCCF treatment in 1974, their utilization has declined in many centers due to availability constraints. A 2023 meta-analysis reported that coils are the most commonly used EVT modality for dCCF (69.3%), followed by Onyx (31.1%), covered stents (22.2%), N-butyl cyanoacrylate (6.7%), and FDs (4.8%) (11). The pooled overall complete occlusion rate was 92.1%. Specifically, the initial occlusion rate was 77.7%, which increased to a final occlusion rate of 93.1%. The pooled symptom improvement rate was 89.4% and the recurrence rate was 8.3%. The pooled complication rate was 10.9%. Specifically, the most common major complication was cranial nerve palsy (3.3%), followed by intracranial hemorrhage (0.5%).

In our study, the initial complete occlusion rate of 76.9% increased to 100% at FU. No procedural/delayed complications or fistula recurrence was observed. The favorable outcomes were attributed to strict patient selection, optimal stent apposition, and proactive management of endoleak/residual leakage. Notably, visual loss (likely secondary to primary optic nerve injury) was irreversible even after successful fistula occlusion, whereas visual decline showed potential for reversal with timely intervention. These findings aligned with previous reports indicating that pre-existing optic nerve damage was a major determinant of irreversible visual impairment in dCCF.

### Advantages of WCS

The WCS is a balloon-expandable endoprosthesis consisting of a bare stent and an expandable polytetrafluoroethylene (PTFE) membrane, specially designed for use in the tortuous intracranial vasculature (6). Over the past decade, our team has validated its utility as a simplified, effective, and time-efficient treatment for various cranial vascular lesions (12–15). The WCS seemed to be an ideal therapeutic option for two key advantages: (i) immediate parent artery reconstruction without intraluminal mass protrusion, which avoids the risk of vessel occlusion or stenosis; and (ii) preservation of normal CS blood flow and cranial nerve function, as intra-fistulous manipulation is unnecessary, thus reducing the risk of cranial nerve palsy, a common complication of coil or liquid embolic agent use.

### Fistulous rent identification

**Figure 3:**
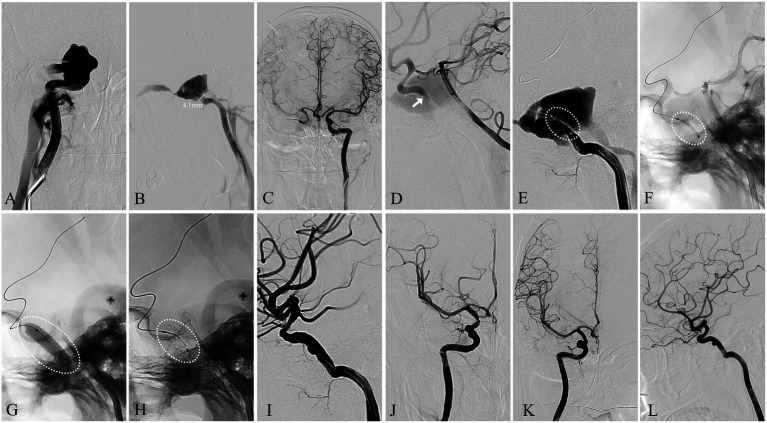
Angiographic images of a 50-year-old man with a right traumatic dCCF (see Patient No. 9 in Table 3). **(A)** A-P view and **(B)** Lateral view of a right-sided traumatic dCCF and the diameter of the parent artery (straight line). **(C)** A-P view of the collateral compensation from the contralateral ICA **(D)** The site of the fistulous rent (arrowhead) was identified at the vertical segment of the cavernous ICA by the vertebral angiography. **(E)** The WCS (dotted oval) 4.5 × 13 mm was delivered in position to cover the fistula. **(F)** The position of the WCS (dotted oval) was reconfirmed by the vertebral angiography. **(G)** Initial balloon inflation (dotted oval) **(H)** The WCS (dotted oval) was deployed. **(I)** lateral view and **(J)** A-P view of the complete occlusion of the dCCF and the preservation of the parent artery. **(I)** A-P view and **(L)** lateral view of the 12-month angiographic FU.

Accurate identification of the fistulous rent is critical for successful dCCF treatment, particularly in high-flow cases. Supplementary angiographic evaluations (vertebral artery and contralateral carotid artery angiography) and 3D rotational angiography facilitated precise fistulous rent identification. As demonstrated in our study, vertebral artery angiography not only aided in fistulous rent identification, but also supported microcatheter navigation and intraoperative monitoring (Figure 3). 3D roadmap imaging was additionally valuable for differentiating dCCF from other lesions, such as ruptured cavernous ICA aneurysms, as illustrated in one of our spontaneous cases (Figure 2). Furthermore, the “broken-rim sign” on MPR imaging orthogonal to the fistula provided a practical and reliable method for localizing and measuring the fistulous rent. Which was essential for optimal stent size selection (Figure 1).

### Technical pearls for WCS delivery and apposition

Despite its intracranial design, successful WCS delivery and apposition in tortuous arteries remain challenges. Fistulous rent location and parent artery tortuosity are major determinants for technical success. First, fistulous rent location impacts stent apposition: dCCFs in straight vascular segments (vertical and horizontal portion of the cavernous ICA, which accounted for 76.9% of cases in our study) are more suitable for WCS placement and apposition than those in curved vascular segments (anterior and posterior bend of the cavernous ICA). In our experience, dCCFs in the posterior bend of the cavernous ICA were eligible only if the bend angle is obtuse (≥ 90°), as seen in 3 enrolled patients (23.1%); acute-angled bend (< 90°) was not recommended due to the risk of inadequate stent apposition and subsequent endoleak. Second, parent artery tortuosity is a major barrier to WCS delivery, which can be overcome with the use of intracranial support catheters. A critical technical pearl is to advance the WCS beyond the fistula within the intracranial support catheter to prevent unintended stent migration during deployment, which significantly improved technical success in our series (16–18).

### Endoleak management

Endoleak is defined as persistent perfusion of the space between the covered stent and parent vessel wall (19). Stent size selection is paramount for minimizing endoleak risk. When in doubt, our experience is to choose a WCS that is larger in diameter to ensure stent apposition and longer in length to secure adequate coverage, owing to the absence of vital branches or perforating arteries in the cavernous ICA (18). Nevertheless, endoleak occurred in 6 patients (46.2%) after initial WCS deployment in our study, attributable to two main factors: (i) insufficient initial balloon inflation (due to over-caution to avoid arterial dissection or rupture, considering the diameter gradients along the covered vessel), which could be resolved by balloon re-inflation with moderately increased pressure; and (ii) inadequate stent apposition on the concave side of curved vessels (an inevitable limitation associated with the rigid structural properties of the covered stents).

For endoleaks caused by insufficient initial balloon inflation (*n* = 5), balloon re-inflation with moderately increased pressure resulted in complete resolution in 4 patients and alleviation to minimal endoleak in 1 patient (Figure 2). For the endoleak (*n* = 1) attributed to inadequate concave apposition, alleviation was achieved but minimal endoleak persisted despite of multiple balloon re-inflations. These two cases of persistent minimal endoleak were consistent with previous reports indicating favorable outcomes for minimal endoleak, particularly in the middle-to-distal stent segments (7, 11, 20, 21).

In contrast, significant distal leakage (distinct from endoleak) secondary to inadequate stent coverage, resulting from incorrect fistulous rent measurement and undersized stent selection, required urgent remedial EVT strategies. In this case, rescue embolization with coils and Onyx was successfully performed.

## Limitations

This study had several notable limitations. First, it was a single-center retrospective study with a small sample size (*n* = 13), which restricted the statistical power of our analyses and limited the generalizability of the findings. Second, the retrospective design inherently introduced selection bias: enrollment criteria were subjectively evaluated by two senior interventional neuroradiologists, and EVT modality decisions were made jointly with patients and their relatives based on clinical preferences and shared decision making. Third, long-term angiographic FU data were limited (mean duration: 9.00 ± 5.87 months), precluding comprehensive assessment of long-term vascular outcomes such as neointimal hyperplasia and in-stent stenosis. Fourth, the absence of a control group (patients treated with other EVT modalities such as coils or FDs) prevented direct comparison of the WCS with alternative treatments for dCCFs.

## Conclusion

Our findings demonstrated that the WCS was a safe and effective therapeutic option for the treatment of dCCFs, with favorable long-term clinical outcomes. Strict patient selection, precise fistulous rent localization, optimal stent apposition, and proactive endoleak management were critical for achieving these outcomes. Nevertheless, given the study limitations, additional large-scale prospective studies and randomized controlled trials are warranted to validate the efficacy and safety of the WCS for dCCFs.

## Data Availability

The raw data supporting the conclusions of this article will be made available by the authors, without undue reservation.
